# Thermal-Temporal Treatment Preparation of the Melt Before Amorphization to Obtain Nanocrystalline Magnetic Cores with Unique Magnetic Characteristics

**DOI:** 10.3390/nano16150922

**Published:** 2026-07-27

**Authors:** Vladimir S. Tsepelev, Kaiming Wu, Nadezhda P. Tsepeleva

**Affiliations:** 1 Research Center for Physics of Metal Liquids, Institute of New Materials and Technologies, Ural Federal University, 19, Mira Str., 620002 Ekaterinburg, Russia; n.p.konovalova@urfu.ru; 2 International Research Institute for Steel Technology, Wuhan University of Science and Technology, 947, Heping Avenue, Wuhan 430081, China; wukaiming@wust.edu.cn

**Keywords:** nanocrystalline soft magnets, liquid–liquid transition, melt pre-conditioning, thermal-temporal treatment

## Abstract

This review presents a current understanding of the relationship between the structure of multicomponent metallic melts and the processes of amorphization and nanocrystallization. Particular attention is paid to the thermal-temporal treatment (TTT) of melts as a precision method for monitoring the nonequilibrium state of the liquid phase, the relaxation kinetics of cluster associations, and liquid–liquid transitions (LLT). The mechanisms by which precrystallization melt treatment affects the homogeneity of the amorphous precursor, the size of nanograins (7–15 nm), the phase composition (Fe_3_Si, Fe_2_B), and the resulting magnetic characteristics of toroidal cores (μ*_max_* > 600,000, *H_c_* < 0.5 A/m) are investigated. Based on an analysis of structural models of metallic melts (cybotactic, quasicrystalline, and quasichemical), it is shown that critical temperatures, viscosity hysteresis, and oscillatory relaxation serve as indicators of melt equilibrium. It is noted that the optimized TTT protocols combined with controlled annealing at 542–572 °C enable the formation of Fe_3_Si nanograins with exceptional magnetic softness. The results open the possibility of discussing the prospects for integrating TTT with in situ diagnostics, CALPHAD modeling, and the potential of machine learning for the design of next-generation soft magnetic nanomaterials with tailored frequency characteristics for high-frequency power electronics and their use in electromagnetic shielding.

## 1. Introduction

Soft magnetic nanocrystalline alloys based on the Fe–Si–B–Cu–Nb system (Finemet type) and their analogs remain critically important materials for high-frequency power electronics, next-generation transformers, and electromagnetic shielding applications [1,2,3,4,5,6]. A number of studies have presented a critical analysis of existing theoretical models (in particular, the random anisotropy model), identifying their fundamental shortcomings in fully describing the magnetic behavior of nanocrystalline alloys [7]. Strategic paths for creating next-generation materials that overcome the traditional tradeoff between ultra-low power loss and high saturation induction for use in high-frequency power electronics have been outlined.

It is well known that a breakthrough in the field of soft magnetic nanocrystalline materials was made by Yoshizawa and co-workers in 1988, who described a Finemet type alloy (Fe–Si–B–Nb–Cu), which after controlled annealing forms an ultrafine-grained structure [8]. Fundamental contributions to the understanding of these materials in subsequent years were made by G. Herzer, A. Hernando, A. Slawska-Waniewska, A. Inoue, J. Gonzalez, and others [9,10,11,12,13]. The main theoretical tool has been the Random Anisotropy Model (RAM), originally developed by Alben et al. in 1978 to describe the ferromagnetic behavior of amorphous materials [12]. Herzer and Hernando successfully adapted RAM for nanocrystalline systems. It has been shown that when the grain size D is significantly smaller than the exchange interaction length L0, the effective magnetic anisotropy is averaged and drops sharply (proportional to D^6^), which provides an ultra-low coercive force [13,14]. An alternative way to develop materials was proposed by R. Hasegawa. He focused on creating amorphous alloys with extremely high iron content, the key parameter of which is to maximize saturation induction (*B_s_* > 1.7 T), in contrast to nanocrystalline Finemet, where the priority is to minimize coercivity and magnetization reversal losses [15,16]. K. Suzuki et al. showed the possibility of combining high values of *B_s_* and μ*_e_* for base alloys based on the Fe–Zr–B *bcc* lattice, which allows us to expect further development as a new soft magnetic material [17].

A fundamental theoretical contribution to understanding coercivity mechanisms in amorphous and nanocrystalline alloys was made by H. Kronmüller and his school. Within the micromagnetic framework, Kronmüller rigorously described the mechanisms of domain wall nucleation and pinning in nanocrystalline ferromagnets, demonstrating that the coercive force is determined not only by the grain size but also by the magnetic properties of grain boundaries and the degree of magnetic disorder at the interfaces [18]. This approach allowed for a quantitative explanation of why in nanocrystalline materials with grain size *D* < *L_ex_* (exchange interaction length), the effective magnetic anisotropy is averaged out, leading to ultra-low *H_c_* values.

In parallel, M.E. McHenry et al. investigated the family of Co-modified Finemet alloys (Fe–Co–Si–B–Nb–Cu system), which enabled a substantial increase in the Curie temperature and extended the operating temperature range of the materials to 500–600 °C [19]. These works laid the foundation for the development of soft magnetic materials operating under elevated temperature conditions, such as in aerospace and power electronics applications.

A special place is occupied by the family of nanocrystalline FeZrB(Cu) alloys (NANOPERM-type), developed by A. Inoue and K. Suzuki [17]. Unlike Finemet, in these alloys the *bcc*-Fe phase is free of silicon, which ensures higher nanograin homogeneity due to the absence of chemical partitioning during primary crystallization. This leads to improved magnetic softness and enhanced thermal stability of the structure.

Data on the formation mechanisms and methods for producing iron-based nanocrystalline alloys exist, demonstrating how precise adjustment of the chemical composition optimizes their nanoscale microstructure. The ability to achieve high saturation induction (*B_s_*) with low reversal losses positions these alloys as key functional materials for future miniature electronics [20].

Replacing traditional pure electrical iron with amorphous and nanocrystalline soft magnetic materials is being explored to overcome performance limitations in high-frequency magnetic circuits and electromagnetic relays. Dynamic tests and wavelet analysis indicate that the use of nanocrystalline alloys (in particular, 1K107B) significantly improves dynamic response, reduces losses, and increases device accuracy [21]. Laminated nanocrystalline magnetic cores are also used to effectively suppress eddy current losses in high-frequency power electronics. The laminated structure has been shown to not only improve magnetic performance at high frequencies but also significantly enhance the thermal stability of the magnetic cores under harsh operating conditions [22]. Most technological protocols for nanostructure control focus exclusively on annealing after amorphization, while melt preparation before crystallization remains a poorly understood area. Current data confirm that the liquid-metal state is not a passive intermediate phase: its microheterogeneity, cluster dynamics, and relaxation kinetics are directly inherited by the amorphous matrix and determine the uniformity of nanograin nucleation [23,24,25,26].

This review systematizes the results of long-term research in condensed matter physics, the methodology of thermal-temporal treatment (TTT) of melts, and its influence on the properties of nanocrystalline magnetic cores. Particular attention is given to critical temperatures, viscosity hysteresis, liquid–liquid structural transitions (LLTs), and their role in the formation of an equilibrium melt before quenching. The goal of this review is to addresses a gap in the literature by linking the fundamental aspects of metallic liquid structure with applied problems in the design of nanomaterials with unique magnetic characteristics.

The behavior of real multicomponent melts based on 3d transition metals cannot be described by the “simple liquid” theory due to directional d-bonds, impurity interactions, and thermal history [27,28,29]. We consider three complementary structural models—cybotactic, quasicrystalline, and quasichemical—and discuss how modern experimental methods (small-angle neutron scattering, in situ X-ray diffraction) confirm the coexistence of several types of clusters in Al–Si, Fe–B, and Fe–Si–B melts near liquidus temperatures [30,31,32]. The dynamics of their dissolution and recombination underlie strategies for controlling the homogeneity of the amorphous precursor [33,34,35].

This paper presents a critical analysis of modern theoretical models (in particular, the random anisotropy model) and identifies their fundamental shortcomings in fully describing the magnetic behavior of nanocrystalline alloys. Strategic pathways are outlined for the development of next-generation materials that overcome the traditional trade-off between ultra-low power loss and high saturation induction for applications in high-frequency power electronics.

## 2. Structural Models of Multicomponent Metallic Melts

The fundamental question is the structure of the melts. Metallic melts, like any thermodynamic systems, should be divided into two groups: nonequilibrium melts, which temporarily retain structural elements of the original phases, and equilibrium melts, whose structure and properties are determined not by their prehistory but by their composition and state parameters. Both are genetically linked to the original crystalline state. The former by nonequilibrium elements of their structure, the latter by the nature of the interatomic bonds and the short-range order formed [23].

**Figure 1:**
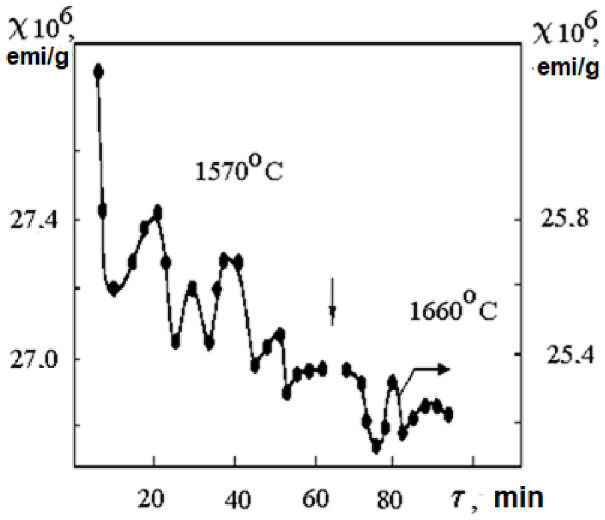
Temporal instability of the magnetic susceptibility χ dependences of the Fe–Cu–O alloy. Adapted from [6].

The free energy of such a nonequilibrium system, *F* = *U* − *TS*, exceeds its equilibrium value, *F_min_*, because the entropy of the ordered regions inherited from the original materials is lower than the equilibrium value, *S_max_*. The motivating factor for studying these, from a thermodynamic point of view, obvious states are well-known experimental facts, such as the dependence [6,23] of the property values of molten samples of practically identical chemical composition on their history (initial materials, heating rates and temperatures), the instability over time of the property values of these melts (Figure 1), the discrepancy between the property values obtained during heating and subsequent cooling of the sample.

The relaxation process of nonequilibrium states, if it occurs at all, often continues for hours. More often, metastable states are established, from which the system cannot spontaneously emerge without thermal or mechanical stimulation. In this case, the simplest means of bringing the melt to equilibrium is additional heating to a fixed temperature.

This threshold nature of the system’s transition to equilibrium indicates a kinetic rather than diffusional regime for this process; i.e., it is most often not the migration of particles that limits the process, but their detachment from the nonequilibrium formation, which requires a relatively high activation energy.

The behavior of real multicomponent melts based on 3d transition metals cannot be described by the “simple liquid” theory due to the presence of directional *d*-bonds, impurity interactions, and thermal history [6,23,29].

Three complementary models are used to describe them:

- The cybotactic model: the melt consists of clusters with short-range order, the boundaries of which are blurred by thermal fluctuations. The cluster lifetime depends on the composition and temperature [23,36].
- The quasicrystalline model emphasizes an ordering tendency similar to BCC/FCC packings, but without the formation of a periodic translational lattice. It allows for the classification of short-range order types in the melt based on geometric parameters, such as the nearest-neighbor distance (*r*_1_) and coordination number (*z*_1_) [36,37]. An important aspect of this model is icosahedral symmetry—a special type of long-range orientational order without translational periodicity, characteristic of the quasicrystalline state. This structure was first discovered by D. Shechtman et al. [38] in rapidly quenched Al–Mn alloys, whose diffraction patterns demonstrated five-fold symmetry, forbidden for classical crystals, caused by the assembly of interconnected polyhedra. The fact that the quasicrystalline state “freezes” upon rapid cooling proves that icosahedral atomic clusters already existed in the liquid phase prior to crystallization. This confirms the concept of structural heredity of the melt and explains the high glass-forming capacity of multicomponent systems.
- The quasichemical model takes into account the unequal nature of interatomic bonds. The most stable clusters are formed by components with the highest interaction energy (e.g., Fe–O, Fe–B, Fe–Si) [29,39]. This model is a development of the views of Stewart, Frenkel, and Eyring [23,36]. This is particularly true for transition metals, since the purest melt of such an element, due to its dual nature, can be considered a special case of this model. Its essence is as follows.

1. The melt consists of microregions—clusters—in which the arrangement of atoms is characterized by a certain order—short-range order.
2. Due to the intense thermal motion of the particles, the clusters do not have clear boundaries. For the same reason, the lifetime of an equilibrium cluster is limited and depends on the composition (the types of chemical bonds) and temperature. The simultaneous existence of clusters with two or more types of ordering is possible.
3. The energetic disparity between interatomic interactions of different types causes the formation of clusters of different structures and compositions, which have different stability over time. The most stable and long-lived clusters are formed by the most strongly interacting components (for example, iron and oxygen).

It is precisely this emphasis on the disparity of interatomic bonds as the cause of microheterogeneity that fundamentally distinguishes this model of microheterogeneous melt structure from other variants, and therefore it is called quasichemical.

Modern experiments (small-angle neutron scattering, in-situ X-ray diffraction) confirm the coexistence of several types of clusters in Al–Si, Fe–B, and Fe–Si–B melts at temperatures close to the liquidus [6,23,32]. The dynamics of their destruction and recombination underlies the control homogeneity of the amorphous precursor [24,30,35,40].

An analysis of these models shows that they do not compete, but rather complement each other, describing various aspects of the structure of multicomponent melts. The cybotactic model elucidates the phenomenon of melt heredity and versions of anomalies of physical properties near the liquidus temperature, but it cannot be applied to multicomponent systems due to ignoring the chemical interaction between dissimilar atoms. The quasicrystalline model provides a description of liquid–liquid transitions and the amorphization mechanism, but requires knowledge of the cluster formation energy, which complicates its quantitative application. The quasichemical model has a rigorous thermodynamic basis, which allows calculating the activities of components and describing systems with negative heat of mixing (Fe–B, Fe–P, Fe–Si). However, it does not take into account topological short-range order. The use of a complex of these models creates an effective methodological basis for targeted control of the structure of an amorphous precursor during the thermal-temporal processing of melts [6,23,29,31,34].

## 3. Liquid–Liquid Transitions and Relaxation Kinetics

Understanding the nature of liquid structures and properties has long been a vibrant research area in condensed matter physics and metallic materials science. The liquid is not homogeneous and the local structures inside change discontinuously with temperature, pressure, etc. The liquid will experience liquid–liquid structure transition under a certain condition. Liquid–liquid structure transition widely exists in many metals and alloys and plays an important role in the final microstructure and the properties of the solid alloys. This work provides a comprehensive review on this unique structure transition in the metallic liquid together with the recent progress of its impact on the following microstructure and properties after solidification. These effects are discussed by integrating them into different experimental results and theoretical considerations. The application of liquid–liquid structure transition as a strategy to tailor the properties of metals and alloys is proven to be practical and efficient [41].

Contrary to intuition, even a single-component substance may have more than two liquid states. The transition between them is called “liquid–liquid transition (LLT).” Recently, we have accumulated evidence for the presence of LLT. This transition may have a strong influence on crystallization, if it exists below the melting point of the crystal. Here we show that crystal nucleation rate can indeed be enhanced by many orders of magnitude near the spinodal point of LLT. This may be used not only for the control of crystalline morphology but also for searching LLT in a metastable state, which is hidden behind crystallization. Our finding also sheds light on how crystallization and other liquid-state transitions can be coupled [42].

The liquid–liquid transition (LLT) in multicomponent melts is an irreversible destruction of microheterogeneous structures inherited from the solid charge [23,24,29]. Its indicators are:

- Viscosity, density, and electrical resistivity hysteresis during heating-cooling;
- Critical temperature (*t_c_*), at which the energy of thermal motion becomes equal to the rupture energy of the strongest interatomic associations;
- Oscillatory relaxation of properties during isothermal holding, indicating the cooperative destruction of dissipative structures [43,44,45,46].

The microheterogeneous state of a chemically heterogeneous melt is understood as the presence of dispersed particles enriched in one of the components, suspended in an environment of a different composition and separated from it by an interphase surface [29]. The microheterogeneous state is destroyed as a result of an energetic impact on the melt, for example, heating to a temperature specific to each composition. After the irreversible destruction of the microheterogeneous state, the melt passes into the state of a true solution, the conditions of its crystallization change, which is reflected in the microstructure, crystalline structure and mechanical properties of the crystallized metal. The concept of the microheterogeneous state of liquid multicomponent alloys was experimentally substantiated by P. S. Popel, U. Dahlborg, M. Calvo-Dahlborg. Using the method of small-angle neutron scattering in melts of Pb–Sn, Al–Si eutectics, regions enriched in one of the elements, separated from the rest of the liquid alloy by a transition layer, were discovered. Two families of particles have been identified: small particles of 10–40 Å in size and large particles of up to 90 Å in size; as the temperature increases, the particles dissolve and recombine into smaller ones [47].

The relaxation kinetics proceed in a kinetic, rather than a diffusion, regime: the activation energy (34–40 kJ/mol) is comparable to the breaking of metal-oxygen or metal-boron bonds [6,45]. Macroscopic stirring does not accelerate the process, whereas holding at *t_c_* for 5–10 min ensures the transition of the melt to an equilibrium state [25,31,33]. Modern studies confirm that LLT can be used as a strategic tool to control the nucleation rate and crystallite morphology, which determine the mechanical and thermal properties of the material [5,6,45,46].

## 4. Thermal-Temporal Treatment (TTT) Pre-Conditioning as a Protocol

There are various methods for bringing a melt to equilibrium. The most readily available method for producing an equilibrium melt is high-temperature treatment. Since the process of bringing a system to equilibrium as a result of heating is non-monotonic and greatly accelerates upon reaching critical temperatures, heating the metal to these temperatures is a key feature of new, advanced technologies. When cooling the liquid metal prepared in this way, its original (non-equilibrium) structure is not restored; i.e., before crystallization, the melt structure is at or near equilibrium and differs significantly from the original.

**Figure 2:**
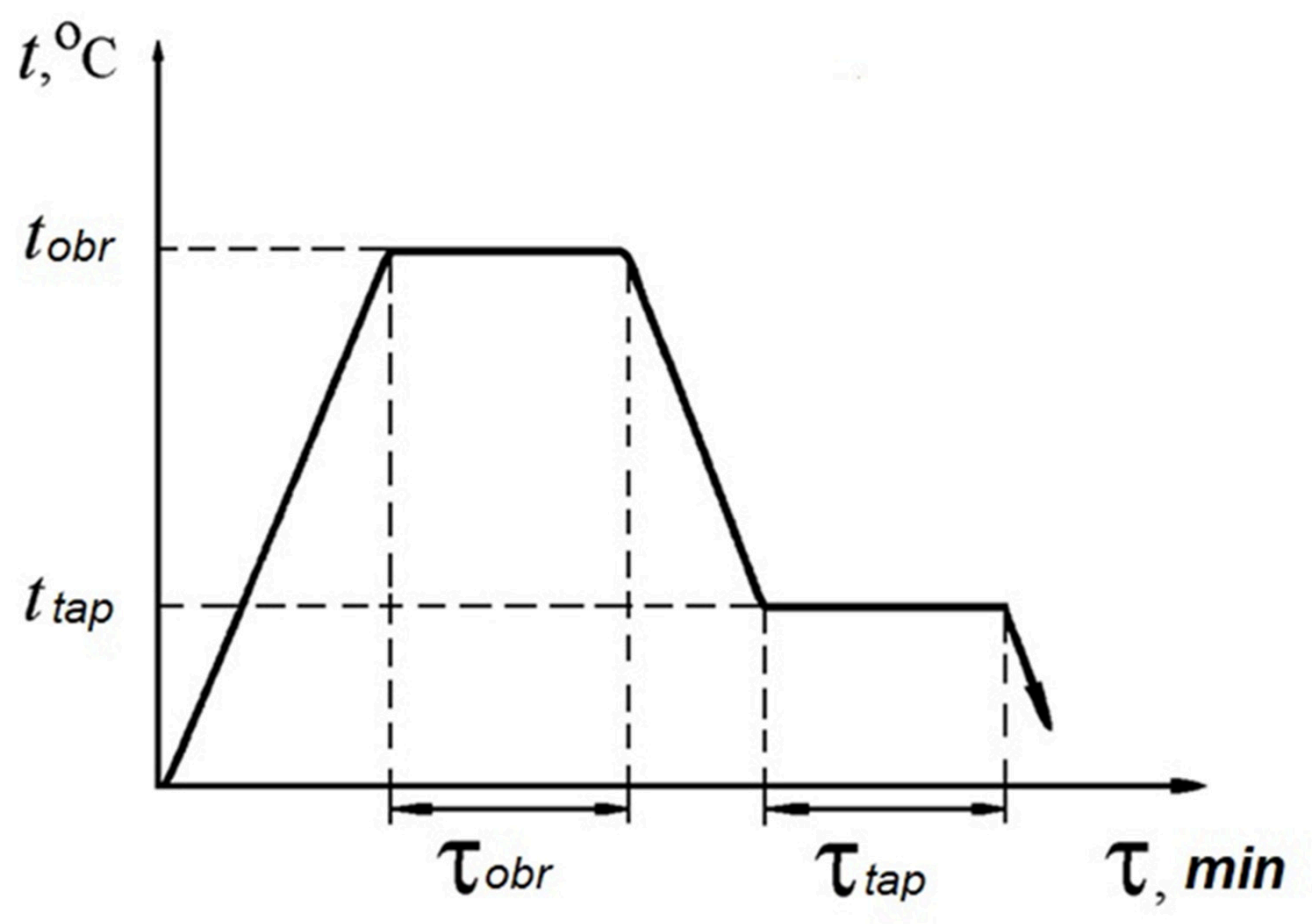
Schematic representation of the thermal-temporal treatment (TTT) protocol for metallic melts prior to amorphization. Key parameters: *t_obr_*—maximum heating. temperature (critical temperature *t_c_* or anomaly temperature *t_an_*); τ*_obr_*—holding time at *t_obr_* (determined by relaxation of viscosity/magnetic susceptibility); *t_tap_*—tapping temperature (typically *T_liquidus_* − Δ*T*, where Δ*T* = 30–80 °C); τ*_tap_*—stabilization hold before casting. Adapted from [6].

The results of numerous experiments and the resulting concepts formed the basis for a promising technological method for managing the quality of metal products through targeted thermal-temporal treatment of the structure of liquid metal. As already noted, this method is called thermal-temporal treatment (TTT) of the melt [23]. In general, the TTT cycle is represented graphically in temperature-time coordinates (Figure 2).

Thermal-temporal treatment (TTT) of the melt is a strictly regulated cycle that ensures the transition of the liquid phase to an equilibrium state before crystallization or amorphization [23,25]. The basic protocol includes:

1. Heating to *t_c_* (or *t_an_* if a temperature anomaly is detected);
2. Holding for τ*_obr_* (determined by the damping of viscosity/magnetic susceptibility oscillations);
3. Accelerated cooling to the tapping/quenching temperature (*t_tap_*);
4. Holding for τ*_tap_* (stabilization before casting).
5. Effective metal stirring allows for a shorter holding time at critical temperatures.

The effectiveness of TTT has been confirmed for over 100 grades of steels and alloys: it reduces chemical inhomogeneity, refines the dendritic structure, and increases plasticity and heat resistance [23,48,49]. In the context of nanomaterials, TTT should be performed immediately before amorphization, since intermediate crystallization and subsequent phase transformations restore the nonequilibrium state of the melt [50,51,52]. Comparison with conventional superheating shows that TTT reduces flow turbulence, improves castability, and increases the thickness stability of the amorphous ribbon [25,31,33].

## 5. TTT as a Precursor for Amorphization and Nanocrystallization

Studies of the physical properties of liquid iron-, nickel-, and cobalt-based alloys intended for the production of amorphous materials deserve special attention. This is due, on the one hand, to high crystallization rates and the preservation of the structural features of the liquid metal in the solid state, and, on the other hand, to the significant influence of kinematic viscosity and surface properties on the technology for producing amorphous ribbons [48].

Alloys with a high content of amorphizers (Fe–Si–B–Cu–Nb–Mo) are characterized by high absolute viscosity values in the pre-solidus region ((14–20)·10^−7^ m^2^/s), which creates technological difficulties during planar flow casting [48].

**Figure 3:**
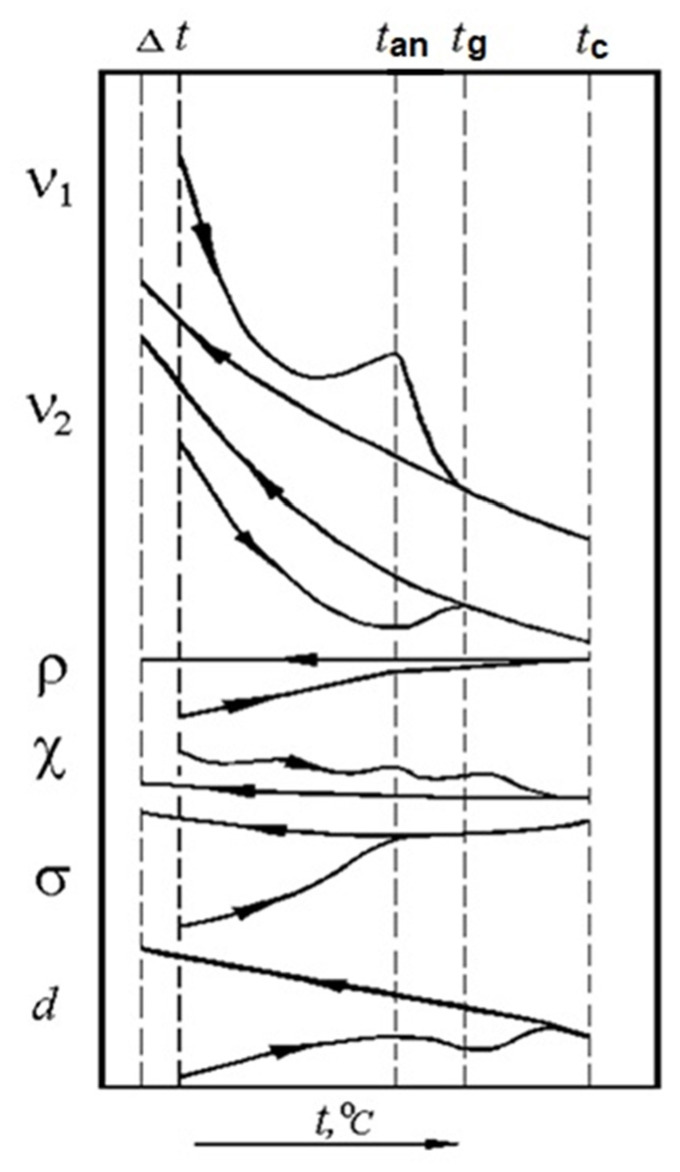
Temperature dependences of physical properties for amorphizing melts temperature dependences of kinematic viscosity (ν_1_, ν_2_—negative and positive hysteresis branches, respectively), electrical resistivity (ρ), magnetic susceptibility (χ), surface tension (σ), and density (d) for Fe-based amorphizing melts (Fe_72.5_Cu_1_Nb_2_Mo_1.5_Si_14_B_9_). Critical temperatures: *t_an_*—anomaly onset, *t_g_*—hysteresis initiation, *t_c_*—critical temperature for melt equilibration; Δ*t*—undercooling degree. Adapted from [6].

A typical temperature dependence of melts with a large number of amorphizing elements is shown in Figure 3.

The critical temperatures *t_c_*, heating to which leads to branching of the polytherms of physical properties, the onset temperatures of hysteresis *t_g_*, and the magnitude of supercooling Δ*t* are determined.

The presence of hysteresis indicates a nonequilibrium initial state of the melt and the need to achieve *t_c_* during the melting process.

In certain cases, it is advantageous to heat the melt only to the anomaly temperature *t_an_*. The required melt holding time at *t_c_* or *t_an_* is determined using the same polytherms and, additionally, by removing the dependence of property values on the isothermal holding time.

The polytherms of viscosity, electrical resistivity, and surface tension demonstrate clear branching at *t_c_*, which correlates with the subsequent homogeneity of nanocrystallization (Figure 3).

**Figure 4:**
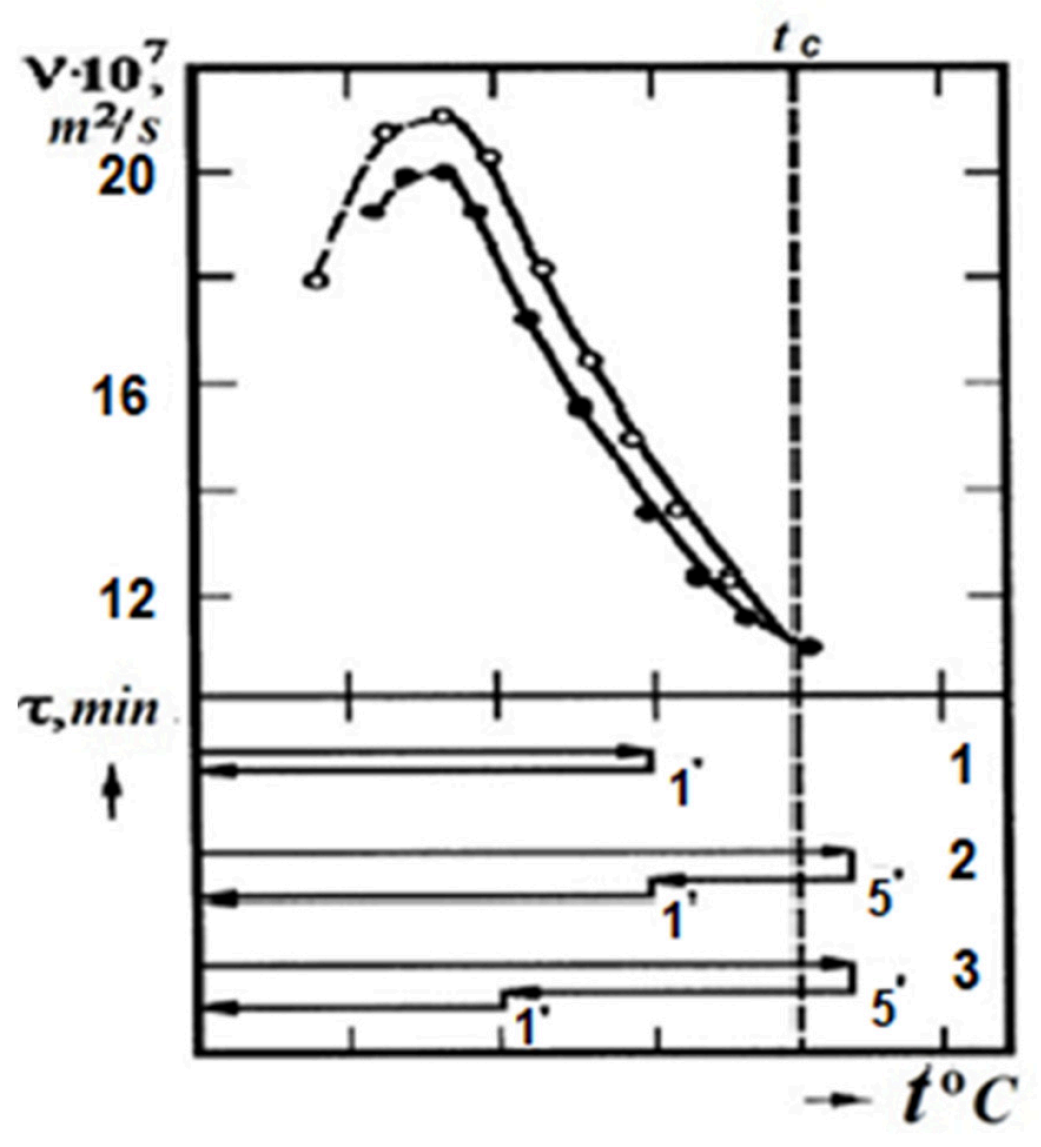
Viscosity polytherms for Finemet-type alloy under different TTT modes quenching. Critical temperature *t_c_* ≈ 1580 °C. Arrows indicate heating (●) and cooling. Kinematic viscosity polytherms of Fe_72.5_Cu_1_Nb_2_Mo_1.5_Si_14_B_9_ melt (Finemet-type) under three thermal histories: (I) heating below *t_c_* + quenching; (II) heating above *t_c_* + quenching; (III) heating above *t_c_* → undercooling → (○) branches. Optimal magnetic properties correspond to Mode III. Adapted from [6].

The kinematic viscosity (Figure 4) of a Finemet-type nanocrystalline magnetic alloy was studied. The first heating regime was to a temperature below *t_c_*, followed by quenching. The second heating regime was to a temperature above *t_c_*, followed by quenching, as in the first regime. The third heating regime was to a temperature above *t_c_*, followed by supercooling and further quenching.

The best performance properties of the ribbons were obtained in the third mode.

The optimal mode includes heating above *t_c_*, holding for 5–10 min, cooling to the liquidus (or below, taking into account Δ*t*), holding for 5 min, and instant quenching [48,52].

The application of TTT allows:

- Destruction of inherited carbide, silicide, and oxide clusters;
- Ensuring a uniform distribution of Cu nuclei in the amorphous matrix;
- Reducing the scatter of properties from ribbon to ribbon.

An analysis of the physical property polytherms of liquid iron-, nickel-, and cobalt-based alloys prone to amorphization reveals the following key patterns. Critical temperatures were established for virtually all the alloys studied. Heating to these temperatures leads to branching of the polytherms of kinematic viscosity, surface tension, electrical resistivity, magnetic susceptibility, and other physical properties. This indicates a nonequilibrium state of the initial melts and the need to achieve a critical temperature during their smelting. Moreover, thermal-temporal treatment of the melt should be performed immediately before producing the amorphous ribbon, rather than at the stage of smelting the blank. Intermediate crystallization of the melt and subsequent solid-phase transformations will again lead to a nonequilibrium state of the melt before its amorphization.

**Figure 5:**
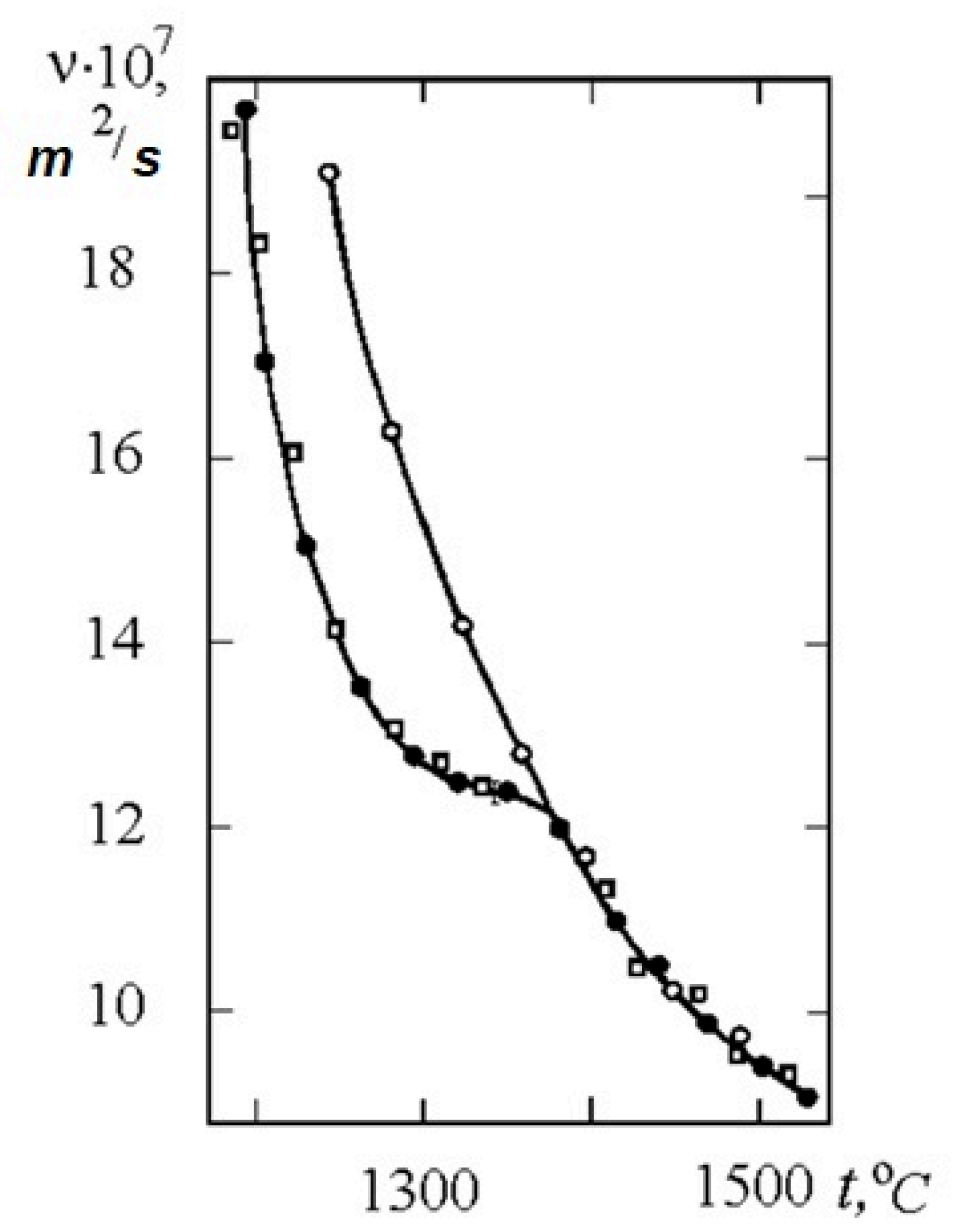
Hysteresis of kinematic viscosity in Ni-based amorphous brazing alloy (VPr-type) demonstrating the irreversibility of melt equilibration. After intermediate crystallization (● → ○ → □), viscosity values revert to the initial heating branch, confirming that TTT must be applied immediately before amorphization, not at the ingot melting stage. Adapted from [6].

Intermediate crystallization nullifies the effect of TTT, which is confirmed by the restoration of viscosity hysteresis (Figure 5) [48,53,54].

## 6. Structure-Property Relationships in Nanocrystalline Magnetic Cores

**Figure 6:**
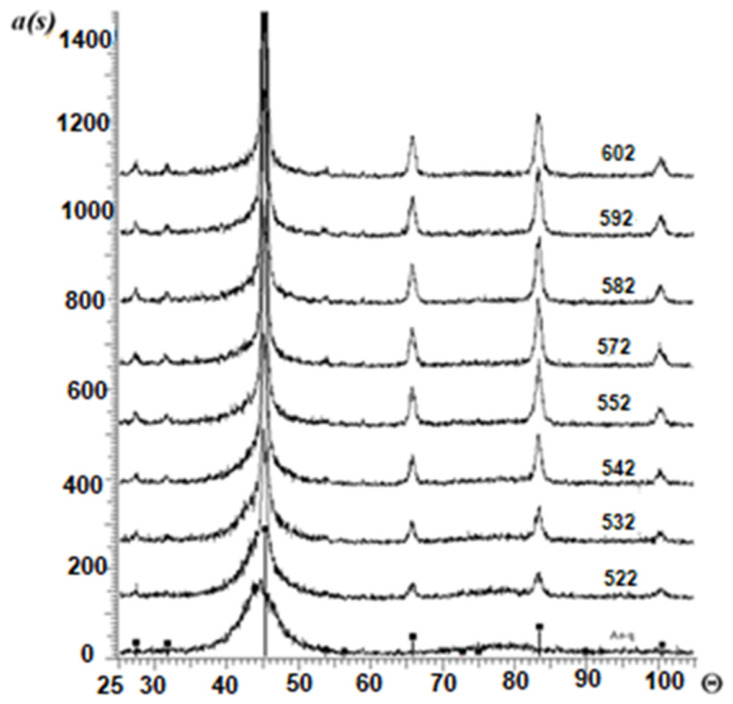
XRD Patterns of nanocrystalline alloy after annealing. X-ray diffraction patterns (Cu-Kα radiation) of Fe_72.5_Cu_1_Nb_2_Mo_1.5_Si_14_B_9_ ribbons after annealing at selected temperatures. As-quenched state shows broad amorphous halo. Crystallization onset at 522 °C: emergence of ordered Fe_3_Si (DO_3_) phase (a = 0.5672 ± 0.0003 nm). Complete nanocrystallization at 542 °C; no amorphous residue detected above 552 °C. Peak indexing: ● Fe_3_Si (111), ■ Fe_3_Si (200), ▲ Fe_2_B (minor phase at Ta ≥ 572 °C). Adapted from [6].

In its initial state, the alloy is X-ray amorphous (Figure 6). The X-ray diffraction pattern shows a diffuse halo without additional reflections from crystalline phases. Crystallization of the alloy upon heating occurs in a single main stage. A single asymmetric exothermic peak is observed on the HTDT curve in the temperature range of 525–600 °C.

After heat treatment at 522 °C, peaks from the crystalline component appear against the background of the amorphous halo. The main crystalline phase is an ordered Fe_3_Si solid solution with a lattice parameter of a = 0.56721 nm. With increasing temperature, the intensity of the halo from the amorphous phase decreases, the intensity of the Fe_3_Si reflections increases, and superstructural reflections become more pronounced. After heat treatment at 542 °C, the alloy is almost completely crystalline—no heat-generating reactions are observed on the HTDT curves.

In the X-ray diffraction patterns of the alloy heat-treated at higher temperatures (552–602 °C), the halo from the amorphous phase is absent, and the lattice parameter of Fe_3_Si does not change within the limits of determination error.

Control of the pre-crystallization melt treatment directly determines the annealing parameters and the final magnetic characteristics. Data for the Fe_72.5_Cu_1_Nb_2_Mo_1.5_Si_14_B_9_ alloy show:

- Optimal annealing: 542–572 °C;
- μ*_max_*: up to 713,000 at 542 °C;
- *H_c_*: minimum 0.41 A/m;
- Grain size: 7–9 nm, with the distribution shifting towards 11–20 nm at *T_a_* > 552 °C.

Magnetic properties of Fe_72.5_Cu_1_Nb_2_Mo_1.5_Si_14_B_9_ nanocrystalline ribbons are a function of annealing temperature (*T_an_*). Samples prepared by planar flow casting (PFC) with TTT-pretreated melt: *t_c_* = 1580 °C, τ*_obr_* = 8 min, cooling rate ≈ 1.2 × 10^5^ K/s, ribbon thickness 22 ± 2 µm. Measurements at f = 1 kHz, *H* = 0.4 A/m.

Table 1 shows the magnetic properties as a function of annealing temperature.

Grain size distribution in Fe_72.5_Cu_1_Nb_2_Mo_1.5_Si_14_B_9_ nanocrystalline alloys is shown after annealing at selected temperatures. Data from TEM analysis (*n* = 215 ± 15 grains/sample). Quenching conditions: melt TTT at *t_c_* = 1580 °C, τ = 8 min; planar flow casting, wheel speed 35 m/s, ribbon thickness 22 ± 2 µm.

The low initial permeability at 522 °C is due to a significant fraction of the amorphous matrix and incomplete nanocrystallization. The increase in *H_c_* at *T_a_* > 572 °C is associated with the precipitation of the magnetically hard Fe_2_B phase [1,51,55].

**Figure 7:**
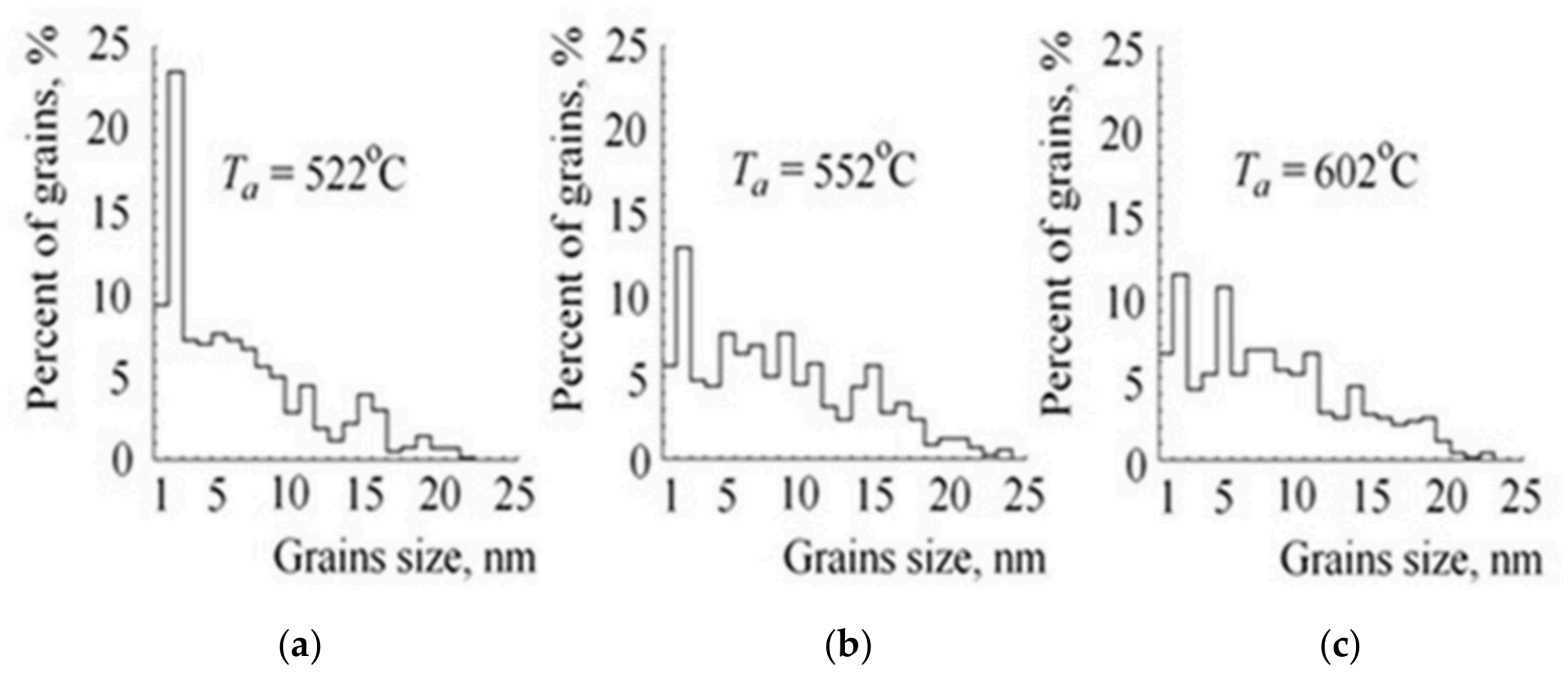
Grain size distribution histograms. Grain boundaries identified via FFT filtering of HRTEM images. Grain size distributions in Fe_72.5_Cu_1_Nb_2_Mo_1.5_Si_14_B_9_ nanocrystalline alloys after annealing at (**a**) 522 °C, (**b**) 552 °C, and (**c**) 602 °C, determined by TEM image analysis (n > 200 grains per sample). Mean grain size: 7 ± 2 nm (522 °C), 9 ± 3 nm (552 °C), 8 ± 3 nm (602 °C). Note the increased fraction of grains >10 nm at higher *T_a_*. Scale bars: 20 nm. Adapted from [6].

Figure 7 shows histograms of grain sizes at different heating temperatures of 522, 552, and 602 °C.

The average grain size remained virtually unchanged at these temperatures, equaling 7, 9, and 8 nm, respectively. However, the grain size distribution changed with increasing temperature. At a low temperature of 522 °C, a significant maximum of 2-nm grains was observed.

These grains, which lack distinct boundaries, are likely nuclei (clusters) of the crystalline phase in the amorphous matrix. At temperatures of 552 °C and 602 °C, the grain size distribution was virtually identical. Moreover, at the higher temperature compared to 522 °C, the proportion of grains larger than 10 nm increased from 20.5% to 33.5% (Table 2).

In the nanocrystalline magnetic alloys Fe_72.5_Cu_1_Nb_2_Mo_1.5_Si_14_B_9_, the low initial permeability corresponds to a structure whose proportion in the residual uncrystallized amorphous matrix is significant. The coercivity of the alloy at an annealing temperature of 522 °C is very low. In this region, the low coercivity does not lead to high initial permeability. The highest initial permeability occurs when the structure is characterized by the largest volume of the ordered Fe_3_Si phase. The increase in the coercivity and the decrease in the initial permeability at high temperatures are explained by the formation of the magnetically hard Fe_2_B phase [51].

Modern analysis confirms that the homogeneity of the amorphous precursor, ensured by TTT, minimizes local stresses, reduces the density of domain wall defects, and ensures the reproducibility of μ and *H_c_* from batch to batch [26,56,57,58].

From the perspective of Kronmüller’s micromagnetic theory [18], the ultra-low coercive force in nanocrystalline soft magnetic alloys is explained not only by the small grain size (D ≈ 7–15 nm) but also by the optimal ratio of magnetic parameters between the crystalline phase and the amorphous intergranular layer. According to Kronmüller’s model, the effective magnetic anisotropy constant Keff is averaged over the exchange interaction volume and is proportional to K1^2^·D^6^/A^3^, where K1 is the magnetocrystalline anisotropy constant and A is the exchange stiffness constant. This explains why FeZrB(Cu) alloys with a more homogeneous nanograin structure (absence of Si in the bcc phase) achieve lower coercivity compared to Finemet at comparable grain sizes.

McHenry’s studies on Co-rich alloys [19] demonstrated that the temperature dependence of magnetization M (T) in nanocrystalline materials is governed by two factors: (i) the temperature dependence of the amorphous intergranular layer magnetization, which decays faster than in the crystalline phase, and (ii) the exchange coupling between grains through the amorphous layer, which weakens as the temperature approaches the Curie temperature of the amorphous phase. This explains the existence of an optimal Co content at which the maximum operating temperature is achieved without loss of magnetic softness.

## 7. Challenges, Scalability, and Future Perspectives

Despite its proven effectiveness, the implementation of TTT in the industrial production of nanocrystalline ribbons faces several barriers:

- The lack of in-situ viscosity and density monitoring systems in real-time on melting installations;
- The energy consumption of long isothermal holds;
- The difficulty of scaling protocols for high-entropy and Co/Ni-based alloys.

Future perspectives include:

- Integration of TTT with magnetic pulse and ultrasonic melt processing [3,59];
- Application of CALPHAD and molecular dynamics modeling to predict *t_c_* and τ*_obr_* [4,42];
- Use of machine learning to optimize temperature-time windows for specific chemical compositions [60,61];
- Development of in-situ diagnostics (synchrotron X-ray, neutron scattering) for direct visualization of LLT and cluster dynamics [24,27,41].

The productivity of the integrated approach is confirmed by specific examples. For example, in the work of Sun et al. [61], the XGBoost algorithm, trained on a data set (approximately 500 compositions of Fe-based alloys), was used for preliminary calculation of the saturation induction *B_s_*, coercivity *H_c_*, grain size, and the width of the annealing temperature-time window Δ*T_x_*. The calculation error was 2.3–9.4%. This allowed the researchers to find and describe the Fe_85.5_B_11_P_1.5_Cu_0.5_Nb_1.5_ alloy with a record-wide annealing window Δ*T_x_* = 168 K; its experimental verification confirmed the achievement of *H_c_* = 5.15 A/m and *B_s_* = 1.76 *T*. As we can see, the use of molecular dynamics methods in combination with CALPHAD modeling enables the preliminary prediction of critical temperatures *t_c_* and holding times τ*_obr_* for specific compositions, which significantly increases the efficiency of developing new soft magnetic alloys while reducing the amount of experimental work [4,41,42]. The introduction of computational methods into in situ synchrotron diagnostics opens the prospect of creating “digital twins” of the thermal-time processing process, enabling the adjustment of melt parameters in real time to produce nanocrystalline magnetic cores with specified frequency characteristics.

This review summarizes the results of the authors’ long-term research in the field of condensed matter physics. It analyzes current trends in the study of metallic liquids at high temperatures, the structure of multicomponent metallic melts, and specific methods for their analysis. The principles of thermal-temporal treatment of melts are presented. Particular attention is paid to the relationship between the liquid and solid states of metals. The essence of advanced technologies for producing amorphous and nanocrystalline materials is revealed [62,63].

## 8. Conclusions

1. Thermal-temporal treatment (TTT) of metallic melts represents a physically grounded methodology for controlling the nonequilibrium state of the liquid phase, thereby ensuring homogeneity of the amorphous precursor and reproducibility of nanocrystallization. The protocol involves heating to critical temperatures (*t_c_*), isothermal holding (*τ_obr_* = 5–10 min), controlled cooling to tapping temperature, and stabilization prior to casting.
2. Critical temperatures, viscosity hysteresis, and relaxation kinetics serve as key indicators of achieving melt equilibration prior to amorphization. The irreversible nature of structural transformations during heating—evidenced by branching polytherms of viscosity, electrical resistivity, and magnetic susceptibility—confirms the elimination of inherited nonequilibrium clusters.
3. An optimized TTT protocol combined with annealing at 542–572 °C enables the formation of Fe_3_Si nanograins with a mean size of 7–9 nm, achieving maximum relative permeability *μ_max_* > 600,000 and coercive force *H_c_* < 0.5 A/m. The narrow grain size distribution (*σ_d_* = 3–4 nm) and minimal fraction of magnetically hard Fe_2_B phase are critical for superior magnetic softness.
4. Integration of TTT with modern computational materials science methods (CALPHAD, molecular dynamics), in-situ diagnostics (synchrotron X-ray, neutron scattering), and AI-driven optimization opens pathways for designing next-generation nanocrystalline magnetic cores with tailored frequency response and core loss characteristics for high-frequency power electronics operating in the 1.5–380 GHz range.

## Figures and Tables

**Table 1:** Magnetic properties as a function of annealing temperature.

*T_a_* (°C)	μ_0.08_ (±5%)	μ*_max_* (±8%)	KR (±0.03)	*H_c_* (A/m) (±0.05)	Dominant Phase (*s*) Comp.	Finemet [34] μ*_max_* Comp.	Nanoperm [7] *H_c_*
482	7300	147,000	0.69	2.15	Amorphous + Fe_3_Si nuclei	120,000 (*T_a_* = 550 °C)	0.8 A/m (*T_a_* = 500 °C)
502	14,400	330,000	0.77	1.25	Fe_3_Si (minor)	—	—
522	25,000	394,000	0.65	0.90	Fe_3_Si + amorphous	—	—
532	35,000	540,000	0.63	0.66	Fe_3_Si (major)	—	—
**542**	**52,000**	**713,000**	**0.63**	**0.41**	Fe_3_Si (fully nano)	**650,000** (optimal)	**0.5** A/m (optimal)
552	91,000	663,000	0.59	0.45	Fe_3_Si + Fe_2_B trace	—	—
562	98,000	664,000	0.63	0.46	Fe_3_Si + Fe_2_B trace	—	—
572	120,000	688,000	0.61	0.51	Fe_3_Si + Fe_2_B	—	—
582	105,000	588,000	0.58	0.56	Fe_3_Si + Fe_2_B	—	—
592	69,000	430,000	0.56	0.56	Fe_3_Si + Fe_2_B	—	—
602	61,000	237,000	0.59	1.58	Fe_3_Si + Fe_2_B (coarsened)	—	—

Notes: μ_0.08_—initial permeability at *H* = 0.08 A/m; μ*_max_*—maximum permeability; KR—squareness ratio (Br/Bs); *H_c_*—coercive force. Optimal properties highlighted in bold. Literature data: [33] Tsepelev and Starodubtsev, Nanomaterials 2021; [7] G. Kumar, T. Ohkudo, and K. Hono. Journal of Materials Research 2009. Adapted from [6].

**Table 2:** Grain size distribution after annealing.

*T_a_* (°C)	Mean Size *d* (nm)	σ*_d_* (nm)	0–5 nm (%)	6–10 nm (%)	11–15 nm (%)	16–20 nm (%)	21–25 nm (%)	>25 nm (%)	Fe_2_B Fraction *
522	7 ± 2	3.1	54.6	27.4	13.5	6.1	0.9	<0.5	—
552	9 ± 3	4.2	35.4	30.4	21.2	9.7	2.6	0.7	~2 vol%
602	8 ± 3	4.5	37.8	29.5	19.4	11.0	2.4	0.9	~5 vol%

* Estimated from XRD Rietveld refinement; Fe_2_B is magnetically hard phase (*H_c_* > 50 A/m). Comparison: Finemet typical grain size 10–15 nm [26]; Nanoperm 8–12 nm [7]. TTT-pretreated samples show narrower size distribution (lower σ*_d_*) as conventional melting. Adapted from [6].

## Data Availability

The original contributions presented in this study are included in the article. Further inquiries can be directed to the corresponding author.

## Abbreviations

The following abbreviations are used in this manuscript:

TTT	Thermal-temporal treatment
LLT	Liquid–liquid transition
*t_c_*	Critical temperature for melt equilibration
τ*_obr_*	Isothermal holding time at *t_c_*
μ	Relative magnetic permeability
*H_c_*	Coercive force
Finemet-type	Fe–Si–B–Cu–Nb based nanocrystalline soft magnetic alloy
